# Reasons for Outdoor Tanning in Adults: Q Methodology Identifies Three Types of Tanners

**DOI:** 10.1155/jskc/5592331

**Published:** 2025-05-06

**Authors:** Ciara Bergmann, Tobias Konkel, Tatiana Görig, Esma Dursun, Katharina Diehl

**Affiliations:** ^1^Professorship of Epidemiology and Public Health, Department of Medical Informatics, Biometry and Epidemiology, Friedrich-Alexander-Universität Erlangen-Nürnberg (FAU), Erlangen, Germany; ^2^Bavarian Cancer Research Center (BZKF), Erlangen, Germany; ^3^Comprehensive Cancer Center Erlangen-European Metropolitan Area of Nürnberg (CCC ER-EMN), Erlangen, Germany

**Keywords:** Germany, prevention, Q methodology, reasons, skin cancer, sunlight, tanning, ultraviolet radiation

## Abstract

**Background:** Ultraviolet (UV) radiation from the sun is a major risk factor for skin cancers. Nonetheless, many individuals in western countries tan outdoors. This study aimed to identify types of tanners and their reasons for outdoor tanning based on a Q methodology study.

**Methods:** A heterogeneous sample of 25 participants aged 19–61 years was recruited and interviewed using the Q sort method. The participants ranked 37 reasons for outdoor tanning according to their subjectively perceived importance in a predetermined grid. Data were analyzed using an inverted factor analysis technique developed specifically for Q methodology. The transcripts of the postsort interviews were used to better understand the quantitative findings.

**Results:** Three groups (factors) of tanners were identified based on 22 of the 25 participants. While enhancement of attractiveness was a major reason for outdoor tanning in the middle-aged group, the youngest and the oldest groups had a stronger focus on relaxation, well-being, and mental health. All three groups stated that vitamin D production was an important factor for tanning outdoors.

**Discussion:** These findings suggest that people may have different reasons for exposing themselves to harmful UV radiation, but that there is a pattern most participants in our study could be assigned to. Especially the finding that all groups tanned outdoors to meet their vitamin D needs seems to be a starting point for future research and skin cancer prevention.

## 1. Introduction

The major environmental risk factor for the development of skin cancer is ultraviolet (UV) radiation, which can damage the DNA of skin cells [1]. According to the World Health Organization (WHO), one in every three diagnosed cancers is skin cancer [2]. For example, in Germany, the age-standardized incidence of malignant melanoma, the most dangerous type of skin cancer, tripled between 1980 and 2004 [3] and is still increasing [4]. In 2022, the Global Cancer Statistics (GLOBOCAN) estimated 331,647 new cases of malignant melanoma and 1,234,595 new cases of nonmelanoma skin cancer (NMSC) worldwide [5].

The most important source of UV radiation for the general population is sunlight. However, UV radiation can also be generated artificially as found in tanning beds [6]. Since tanning beds have been classified as carcinogenic to humans, some countries have implemented legislation, for instance, to protect minors [7].

Despite health warnings, many people in western countries expose themselves to natural and artificial UV radiation. Although there have been several studies on the reasons for using tanning beds, the reasons for intentional outdoor tanning have been neglected in previous research. The most frequently named reasons for using tanning beds were enhancing attractiveness, achieving a state of relaxation, feeling light and warmth, and medical reasons (e.g., skin diseases) [8–11]. Previous studies on the motivation for tanning outdoors have focused mainly on different aspects of attractiveness (e.g., feeling skinnier, having more muscle tone, and more sex appeal), the role of family and friends, and media [12–15]. In addition, the focus has been mainly on adolescents and students [12, 13, 16], although studies have shown that middle-aged people in particular tan outdoors [17].

Therefore, our aim was to identify subjective views regarding potential reasons for intentional outdoor tanning in adults. We used the innovative Q methodology, a combination of qualitative and quantitative data, to identify combinations and interrelations of potential reasons for outdoor tanning by the participants (so-called viewpoints) [18, 19]. Sorting and ranking items using the Q methodology allowed us to obtain rankings on reasons for tanning based on the individuals' “psychological significance” and the perceived meaningfulness [20]. By using this method, we were able to not only identify the most important reasons for intentional outdoor tanning but also provide information on why this is the case and who shares these views. The identification of groups of outdoor tanners with similar attitudes and characteristics is important for future prevention.

## 2. Methods

We conducted a Q methodology study among 25 individuals who stated that they intentionally tanned outdoors. The study comprised a classic card sorting activity (Q sort), a postsorting interview, and a quantitative questionnaire. The fieldwork was performed by the first author (CB, medical student), who was trained in conducting interviews before the first interview by the last author, an experienced qualitative researcher (KD, public health researcher and social scientist). All participants provided written informed consent to participate in this study. The study was approved by the Ethics Committee of Friedrich-Alexander-Universität Erlangen-Nürnberg on December 12, 2022 (22-412-S).

### 2.1. Participants

Twenty-five participants (mean age: 38.3 years, 52% female) took part in the study. The inclusion criteria were age 18–65, intentional tanning outdoors, and proficiency in German. Participants were recruited through a convenience sample via different channels, including a study flyer, a call for participation on the webpage of the professorship, and social media. We aimed to obtain a heterogeneous sample that included different ages (age range: 19–61), education levels (high, medium, and low), and a nearly equal distribution of men and women. All participants received a 15€ gift voucher after the interview as compensation for their time. The interviews took place face-to-face between May 23, 2023, and September 6, 2023.

### 2.2. Procedure

Q sort is a sorting and ranking procedure used for researching subjective opinions, value structures, and attitudes toward various issues [21, 22]. It was invented in the 1930s by William Stephenson, a psychologist and physicist, and has evolved over the years [20].

**Figure 1:**
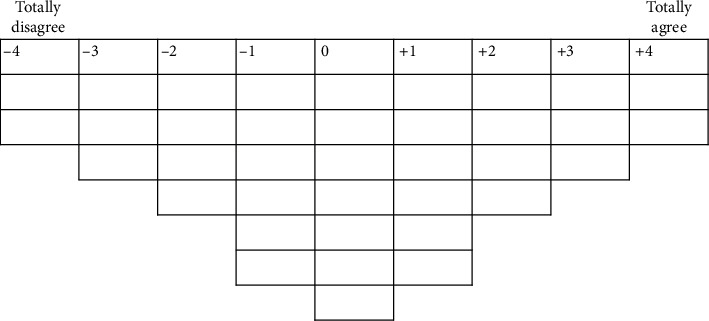
Grid used for Q sort. Legend: Participants sorted the 37 items along the individually perceived agreement.

After completing a short questionnaire on sociodemographic characteristics, the interviewer (CB) explained the Q sort procedure to the participants. They were asked to order a set of printed cards (the so-called Q sort deck) consisting of 37 items comprising various reasons for tanning outdoors (Table 1) on a Q sort grid (the so-called forced distribution, Figure 1). Items were developed based on previous studies on outdoor and indoor tanning [12, 19, 23–30] and brainstorming by the authors. The grid (Figure 1) showed the distribution of items resembling a quasi-normal distribution with extreme values of +4 (totally agree) and −4 (totally disagree) and a medium category (0). In the first step, the participants presorted the 37 items into three piles: agreement, neutral, and disagreement. In the second step, the cards were placed from the three piles, one after the other, on the grid. During this sorting procedure, the think-aloud method was used to determine the participants' subjective viewpoints. The interviewer assisted the participants when they were in doubt during the sorting process. The completed Q sort grids were photographed.

Subsequently, postsorting interviews were conducted. The average duration was 34 min (min: 22, max: 47). The interviews were audio-recorded, transcribed verbatim, double-checked, and analyzed by two independent scientists (CB and ED). The interviews were used to explain the participants' perceived importance of items.

### 2.3. Analysis

Data from the Q sort grids were digitalized and analyzed based on an inverted factor analysis developed specifically for Q methodology [19, 30]. Unlike traditional factor analysis, in which participants' responses to a number of variables or items are correlated (i.e., by-variable), Q factor analysis (i.e., by-person) tests the associations between participants, resulting in a number of factors that cluster participants who have ordered their cards similarly on the Q sort grid [18].

For the Q factor analysis, we used the statistical program R using the package qmethod [31]. We used principal component analysis (PCA) to extract the factors [19]. The number of factors chosen was based on the following suggestions by Watts and Stenner [19]. Factors needed to have an eigenvalue greater than 1.00 and at least two Q sorts (i.e., two persons) that load significantly on a single factor. We used varimax rotation to maximize the variance explained by the factor solution.

Factors are described based on transcripts obtained from the postsorting interviews and the analysis of the sociodemographic data from the questionnaire. For the latter, IBM SPSS for Statistics (Version 27) was used for descriptive analysis.

## 3. Results

The Q factor analysis revealed a solution with three factors that satisfied the criteria described in the Methods section (Table S1). This factor solution was defined by 22 out of 25 participants who loaded on a single factor. Three participants (P01, P11, and P23) did not significantly load on any factor. The three factors were able to explain 70% of variance (Factor 1: 28%, Factor 2: 25%, and Factor 3: 17%; Table S1).

### 3.1. Demographic Information of Factors

Factor 1 consisted of ten individuals of which seven were female (Table 2). This factor (group) was the youngest one with a median age of 23.5 years. Factor 2 included eight individuals, with a higher proportion of men than women (5 vs. 3), and a median age of 56.5 years. Four individuals (*n* = 1 female) loaded on Factor 3. The median age for this factor was 32.5 years.

Due to the design of the study and the sample size, it is difficult to describe the factors based on the individuals' characteristics in detail. However, to provide an insight into the individuals' tanning and sun protection habits, the frequency of outdoor work, outdoor tanning, and sunburn frequency are presented in Table 2.

### 3.2. Factor Interpretation

Eight of the 37 items were consensus statements, i.e., when factors did not differ significantly from each other in their rating (Table S2). These items covered aspects such as the opinions of significant others and social media (items 17, 22, 26, 27, and 28), a perceived healthier or more athletic appearance made possible by a tan (items 12 and 13), and vitamin D production as a reason for tanning (item 32). While the tan of celebrities (−3 to −2) was less likely to be a reason for tanning in all three groups, participants were more likely to agree that vitamin D production was a reason for tanning outdoors (+2 to +3). The other consensus statements scattered between 0 and ± 2.

Tanned celebrities as a potential reason for sunbathing were not rated important for any of the three groups. Participants stated that they “don't really care about famous people” (P23) or are “not interested in celebrities” (P01). They reported that they generally do not “see celebrities as role models” (P09), “emulate celebrities” (P20), “base [themselves] on celebrities” (P12), and “look at celebrities” (P06). P21 wondered “why so many try to imitate” celebrities. It was also mentioned that it does not matter whether celebrities are tanned because this is “not a attribute you pay any conscious attention to” (P25), “I don't care if they are tanned” (P15), and “when I look up to celebrities, it's for other reasons and not their skin” (P07). Others underlined that they do not find the tan of celebrities attractive or worth having (e.g., “And I often find it rather disgusting how tanned they sometimes are. How artificially tanned and I also think that it makes many people look older.” [P23]; “Because it looks very unnatural.” [P08]).

Not only did Q sort rating reveal that vitamin D production was an important reason for all three groups but also the interviews themselves. The participants reported that “vitamin D deficiency [was] detrimental to the immune system. And that's why, if you work indoors and don't get out that often, you try to compensate for it somehow” (P10). Also, P17 underlined that “you know that vitamin D is good for you and that's why I try to spend a lot of time in the sun.” An important source of knowledge about vitamin D was the media (P01). However, knowledge gaps have also become apparent. For instance, P06 said “without knowing what vitamin D does, but […] vitamin D is good for your health” and P08 expressed uncertainty “I have to say, unfortunately, I'm not very familiar with it [i.e., vitamin D]. I've tried to inform myself before, […] but you always hear different opinions […]. You should go out in the sun so much or that's enough and so on and it's important to me, but I couldn't say what's too much, what's too little.” Some participants were afraid of vitamin D deficiency:

P07: “I want to avoid getting a deficiency and I do not want to eat food supplements, so I go out in the sun on my own.”

P02: “And when it gets to winter, in the peak months, from December onwards, you're in a state of exhaustion and you know that by then your vitamin D will be depleted. And you can actually take a lot of supplements then, but you can't manage it, you can't manage it at all.”

P15: “Vitamin D is important for bones. And various deficiency symptoms, such as tiredness, fatigue, and so on, can all be influenced by vitamin D deficiency. And in summer, as I work in a medical field myself, I always tell our patients to get plenty of sun exposure.”

P02 was even willing to accept the health risks resulting from sun exposure for sake of vitamin D supply: “And that's why the more, the better. […] So you certainly can't avoid every sunburn, but the sun does you more good than harm.”

**Figure 2:**
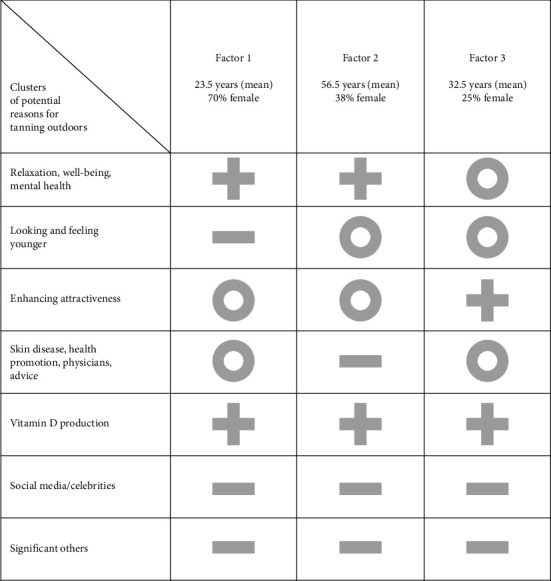
Summary of the viewpoints of the three factors. Legend: Summary based on Q sort estimates. +: relevant; 0: neutral rating; –: not relevant for outdoor tanning.

In the following, the three identified factors (=groups of individuals) will be described in more detail:

 
**Factor 1:** The participants who loaded on Factor 1 shared the viewpoint that relaxation, well-being, and health-related aspects (i.e., vitamin D supplementation, and mental health) were their main reasons for tanning outdoors (item 01: +2; 02: +3; 03: +4; 04: +2; 32: +3; 34: +4; 37: +3). These participants described: “I have the feeling that I am relaxed and have more energy afterwards and feel good” (P04), “Because it's refreshing, the warmth. It warms you up and makes you feel good and you might forget a lot of your worries” (P05), “I also feel a bit […] fitter, from a mental point of view” (P03). Looking and feeling younger, the appearance of celebrities, the impact of social media, or significant others were not important for these participants, while enhancing attractiveness and health aspects besides vitamin D production were rather rated as neutral (Figure 2).
 
**Factor 2:** While those participants who loaded on Factor 2 resembled those loading on Factor 1 in terms of agreement on tanning effects on relaxation and well-being, health reasons (other than mental health and vitamin D) were less important (item 29: −3; 30: −4; 33: −4). In the interviews, all participants who loaded on Factor 2 (P08, P12, P14, P15, P16, P19, P22, and P24) indicated that they did not have any skin diseases, such as acne or psoriasis, and that there was no need to conceal their skin. In addition, most of these participants reported that they were never advised to tan by a physician (P09, P12, P14, P15, P16, P22, and P24). In contrast, they pointed out that outdoor tanning can have negative health consequences, such as the development of skin cancer (P14, P15, P16, and P24). For example, “I can't imagine that physicians encourage people to lie in the sun. Because then, despite everything, if you do it too much, it's simply associated with diseases, right?” (P16). Looking and feeling younger (items 7 and 8) were sorted into the neutral category of the grid (0), while participants loading on Factor 1 showed stronger disagreement with this reason (−2). The difference between Factor 2 and Factor 1 regarding these two items was statistically significant (*p* < 0.001 and *p* < 0.01, respectively; Table S2).
 
**Factor 3:** Participants who loaded on Factor 3 shared the viewpoint that attractiveness-related reasons were their main motives for sunbathing (especially items 10: +2; 11: +4, 15: +3, 18: +4; significant differences to Factor 1 and 2; Table S2). For instance, P20 stated he tanned “because [a tan] just makes the skin look a bit smoother, healthier, more even,” and P25 mentioned “you simply feel more comfortable in your own skin and therefore also more attractive.” Although significant others were not rated as reasons for outdoor tanning, the interviews revealed a connection between the enhancement of attractiveness and the perception of others (for example, “You want to please and, above all, please yourself in the mirror. […] to be noticed by others, to stand out from the crowd in a positive manner. […]” [P18]). Compared to participants who loaded on Factors 1 and 2, all items related to relaxation and well-being (items 01 to 04, 34, 37) were rated significantly lower by the Factor 3 group (all items *p* < 0.001 for both Factor 1 and 2; Table S2). Similar to Factor 2, looking and feeling younger were rated higher than in the Factor 1 group (*p* < 0.001 and *p* < 0.01, respectively; Table S2). In addition, tanning since childhood (item 24) was ranked less important by participants loading on factor 3. This statement was ranked significantly lower (−3) compared to Factors 2 and 3 (both *p* < 0.001; Table S2).

## 4. Discussion

Our Q sort analysis revealed three groups of tanners according to their motivation for outdoor tanning. For all three groups, vitamin D production was an important reason for tanning outdoors. While the youngest (Factor 1) and the oldest groups (Factor 2) named mainly relaxation, well-being, and mental health as motivators, the medium-aged group (Factor 3) had a stronger focus on enhancing attractiveness.

In addition, we identified expected findings regarding age: The youngest group did not attach importance to aspects such as “looking younger” or “feeling younger,” while the oldest group did not rate skin diseases, such as acne, as being important reasons for tanning. These findings can be interpreted as a form of validation of our data.

Synthesis of vitamin D through the sun was an important reason for tanning in all three groups. However, vitamin D production and its potential benefits, such as reducing the risk for osteomalacia, osteoporosis, and poor muscle strength [32, 33], stand in contrast to the health risks of exposure to sunlight, including skin cancer [34]. Prolonged exposure to sunlight is not required to induce vitamin D synthesis through UV radiation. Depending on individual skin type, geographical area, and season, it is sufficient to expose the face, uncovered hands, and arms for 10–15 min two to three times a week [35]. Therefore, it is important to educate people on how much sunlight is good and necessary to maintain vitamin D levels and what is too much or even harmful [36]. In addition, one should keep in mind that tanning is not the only source of vitamin D. Other forms of maintaining beneficial levels are a healthy diet [37], including foods naturally rich in vitamin D, or supplementation in case of a confirmed deficiency [38].

In contrast to previous studies, our study did not identify celebrities and peer pressure as reasons for tanning. This may be due to the participants' age. Previous studies primarily comprised college students [12, 16], while we also included older individuals, which can contribute to the current state of research.

In general, tanned skin is perceived as attractive and healthy in western countries [39]. Studies have shown that tanned individuals are more likely to be perceived as attractive and therefore be credited with more positive attributes, for example, resulting in higher hiring rates [40]. However, our study found that increasing attractiveness through tanned skin was only important for participants loading on Factor 3, i.e., middle-aged adults. For younger and older participants, reasons related to relaxation and well-being were of higher importance for tanning outdoors. For both tanning reasons—enhancing attractiveness and relaxation/well-being—it would be worth considering alternatives to UV radiation and marketing them to reduce the individual skin cancer risk. While light therapy might be a healthy alternative for relaxation, visiting spas with whirlpools and saunas could be a possible replacement for those seeking warmth. For those aiming to obtain tanned skin, sunless tanning products such as spray tan or self-tanning lotions may be an alternative [41]. However, to date, they are not very popular in Germany [42] and potential risks of sunless tanning products are not yet clear [43, 44].

The main reasons for outdoor tanning identified in our study are similar to those revealed in research on indoor tanning: Previous research showed that reasons most frequently named by tanning bed users were well-being, relaxation, and enhancement of attractiveness [8–11]. Vitamin D supplementation was less frequently reported as a reason for tanning bed use but was considered as important for outdoor tanning by all three groups in our study. However, data from Germany show that the relevance of tanning bed use to improve the vitamin D status as a reason for tanning bed use has increased over time [8, 24].

## 5. Limitations

Although the Q methodology has a long tradition, it is still an innovative method because it has not been used very often in health research. Nonetheless, one has to know that data of Q methodology studies are based only on self-reports and reflect the subjective attitudes of the general population. This was also reflected in the subjectivity regarding the evaluations and interpretations of participants in our study. Since the Q methodology combines qualitative and quantitative data elements, our sample size is comparable to that of qualitative studies. This implies that the data were not representative. However, obtaining representative data was not the aim of this study. We tried to include a heterogeneous sample via quota sampling that included different ages and educational backgrounds, as well as men and women, to cover as many opinions and attitudes as possible. Despite this high level of heterogeneity, it was possible to identify patterns in the data and describe three groups of tanners. It may be beneficial to conduct larger quantitative studies to test the replicability of the identified types of tanners and to describe their tanning and sun protection behaviors in more detail. In addition, it would be helpful to assess the skin type as well as the individual and family history of melanoma and NMSC to better understand the individuals' attitudes. This will help develop targeted prevention measures.

Our study shows that Q methodology is feasible and practicable in the field of health research. However, it is important to have a mindful interviewer who supports during the sorting process, especially when a lot of items are included. In addition, we would recommend using the think-aloud technique during the sorting process, although this is not a requirement of the study design. In our study, the participants were free to choose whether or not to use this method. If, however, participants commented their ranking procedure, it offered even greater insights into their thoughts and thus made the respective result more comprehensible. Moreover, the postsort interviews were shown to be very important to increase the quality of the data.

## 6. Conclusion

While quantitative research tries to generalize findings to wider populations [45], Q methodology aims to highlight the diverse thinking patterns among different groups [46]. Our study identified three groups of “tanners,” highlighting that one-size-fits-all prevention strategy will not be able to reach everyone. To reduce the incidence of skin cancer, we should adopt more individualized approaches. In particular, alternatives such as visiting spas or using sunless tanning products could be a focus in prevention strategies. These treatments could fulfill the wishes for tanned skin and relaxation/well-being, respectively. In addition, education about vitamin D is needed, focusing on how much sunlight is needed to meet the vitamin D needs and the unnecessariness of extensive sunbathing for reaching those.

## Figures and Tables

**Table 1:** Items included in Q sort.

Number	Item
01	I tan because I can switch off well when I'm tanning.
02	I tan because it relaxes me to feel the sun on my skin.
03	I tan because the warmth of the sunlight does me good.
04	I tan because tanning in the sun makes me happy.
05	I tan because I feel more confident with tanned skin.
06	I tan because I feel more comfortable with tanned skin than with untanned skin.
07	I tan because I look younger with tanned skin.
08	I tan because I feel younger with tanned skin.
09	I tan because tanned skin makes me feel closer to my ethnic origin.
10	I tan because then I get compliments on my appearance.
11	I tan because I feel more attractive with tanned skin.
12	I tan because I look more athletic with tanned skin.
13	I tan because it makes my body look more muscular.
14	I tan because it makes me more appealing to my partner.
15	I tan because it makes my skin look more even and clean.
16	I tan because I get freckles.
17	I tan because tanned skin is considered desirable in our society.
18	I tan because it makes me look better.
19	I tan because tanned skin increases my sex appeal.
20	I tan because I look slimmer with tanned skin.
21	I tan because the people around me tan regularly and I want to fit in.
22	I tan because the people around me think a tan is attractive.
23	I tan because the people around me think that tanned skin makes me look healthier.
24	I tan because I have been doing so since I was a child.
25	I tan because my parents taught me from an early age that tanned skin is desirable.
26	I tan because my environment says it is good for my health to do so.
27	I tan because social media gives me the impression that tanned skin is desirable.
28	I tan because celebrities also have tanned skin.
29	I tan because of acne or other skin diseases (e.g., neurodermatitis).
30	I tan because tanned skin conceals my skin disease (e.g., acne).
31	I tan because I look healthier with tanned skin.
32	I tan because vitamin D production through the sun is important to me.
33	I tan because my physician recommended me to go out in the sun.
34	I tan because sunbathing is good for my mental health.
35	I tan because the tanned skin is less sensitive to sunburn.
36	I tan to strengthen my immune system.
37	I tan because the sun gives me new energy.

**Table 2:** Demographic characteristics of participants.

	Overall (*n* = 25)		Factor 1 (*n* = 10)		Factor 2 (*n* = 8)		Factor 3 (*n* = 4)	
	*n*	%	*n*	%	*n*	%	*n*	%
Age								
19-38	12	48.0	7	70.0	2	25.0	2	50.0
39-61	13	52.0	3	30.0	6	75.0	2	50.0
Sex								
Female	13	52.0	7	70.0	3	37.5	1	25.0
Male	12	48.0	3	30.0	5	62.5	3	75.0
Immigrant background								
Yes	4	16.0	2	20.0	1	12.5	1	25.0
No	21	84.0	8	80.0	7	87.5	3	75.0
Education								
Low	5	20.0	2	20.0	3	37.5	0	0.0
Medium	5	20.0	1	10.0	3	37.5	0	0.0
High	15	60.0	7	70.0	2	25.0	4	100.0
Employment								
None	7	28.0	4	40.0	1	12.5	1	25.0
Part-time	7	28.0	3	30.0	2	25.0	1	25.0
Full-time	11	44.0	3	30.0	5	62.5	2	50.0
Outdoor work								
Never	16	64.0	5	50.0	8	100.0	3	75.0
Current/former	9	36.0	5	50.0	0	0.0	1	25.0
Intentional outdoor tanning on holidays								
Never	0	0.0	0	0.0	0	0.0	0	0.0
Rarely/sometimes	12	48.0	4	40.0	3	37.5	3	75.0
(Very) often	13	52.0	6	60.0	5	62.5	1	25.0
Intentional outdoor tanning on weekends								
Never	3	12.0	1	10.0	0	0.0	2	50.0
Rarely/sometimes	14	56.0	6	60.0	4	50.0	1	25.0
(Very) often	8	32.0	3	30.0	4	50.0	1	25.0
Intentional outdoor tanning on working days								
Never	7	28.0	2	20.0	2	25.0	3	75.0
Rarely/sometimes	13	52.0	4	40.0	6	75.0	1	25.0
(Very) often	5	20.0	4	40.0	0	0.0	0	0.0
Sunburn (last 12 months)								
Never	6	24.0	4	40.0	2	25.0	0	0.0
Once	8	32.0	2	20.0	3	37.5	1	25.0
More than once	11	44.0	4	40.0	3	37.5	3	75.0

Explained variance	—		28%		25%		17%	

Eigenvalue	—		6.9		6.2		4.2	

*Note:* Three participants did not significantly load on any factor.

## Data Availability

Data are available from the corresponding author on reasonable request.

## Supporting Information

Table S1: factor loadings of the study participants.

Table S2: item score table.
