# Supplementary material for: Reasons for Outdoor Tanning in Adults: Q Methodology Identifies Three Types of Tanners
Source: J Skin Cancer. 2025 May 6;2025:5592331. doi: 10.1155/jskc/5592331 (PMC12074839; doi:10.1155/jskc/5592331)
Supplement: Supporting Information — Additional supporting information can be found online in the Supporting Information section. [file 5592331.f1.docx]

SUPPLEMENT

Table S1: Factor loadings of the study participants

| **Participants** | **Factor 1** | **Factor 2** | **Factor 3** |
| --- | --- | --- | --- |
| P01 | 0.61 | 0.45 | 0.48 |
| P02 | 0.68* | 0.38 | 0.37 |
| P03 | 0.66* | 0.56 | 0.08 |
| P04 | 0.74* | 0.17 | 0.16 |
| P05 | 0.63* | 0.14 | 0.48 |
| P06 | 0.60* | 0.33 | 0.41 |
| P07 | 0.72* | 0.34 | 0.31 |
| P08 | 0.50* | 0.38 | 0.29 |
| P09 | 0.45 | 0.80* | 0.03 |
| P10 | 0.00 | 0.58 | 0.64* |
| P11 | 0.31 | 0.26 | 0.10 |
| P12 | 0.48 | 0.74* | 0.15 |
| P13 | 0.78* | 0.41 | 0.02 |
| P14 | 0.53 | 0.72* | 0.24 |
| P15 | 0.34 | 0.62* | 0.49 |
| P16 | 0.44 | 0.75* | 0.27 |
| P17 | 0.65* | 0.39 | -0.05 |
| P18 | 0.21 | -0.09 | 0.85* |
| P19 | 0.38 | 0.58* | 0.30 |
| P20 | 0.29 | 0.30 | 0.67* |
| P21 | 0.77* | 0.22 | 0.24 |
| P22 | 0.21 | 0.72* | 0.42 |
| P23 | 0.57 | 0.30 | 0.59 |
| P24 | 0.45 | 0.71* | 0.27 |
| P25 | 0.03 | 0.44 | 0.69* |
| **Number of loading Q Sorts** | 10 | 8 | 4 |
| **Explained variance** | 28% | 25% | 17% |
| **Eigenvalues** | 6.9 | 6.2 | 4.2 |

Asterisks (*) mark significant loadings (i.e., Q-sorts which factor loading is higher than the threshold for p-value < 0.05, and Q-sorts which square loading is higher than the sum of square loadings of the same Q-sort in all other factors.

Table S2: Item scores table

| **Statements** | **Factor 1** | **Factor 2** | **Factor 3** | **1 vs 2** | **1 vs 3** | **2 vs 3** |
| --- | --- | --- | --- | --- | --- | --- |
| 1. I tan because I can switch off well when I’m tanning. | 2 | 3 | 0 |  | *** | *** |
| 1. I tan because it relaxes me to feel the sun on my skin. | 3 | 3 | 1 |  | *** | *** |
| 1. I tan because the warmth of the sunlight does me good. | 4 | 4 | 0 |  | *** | *** |
| 1. I tan because tanning in the sun makes me happy. | 2 | 2 | -1 |  | *** | *** |
| 1. I tan because I feel more confident with tanned skin. | 1 | 1 | 2 |  | ** |  |
| 1. I tan because I feel more comfortable with tanned skin than with untanned skin. | 1 | 1 | 3 | * | *** | ** |
| 1. I tan because I look younger with tanned skin. | -2 | 0 | 0 | *** | *** |  |
| 1. I tan because I feel younger with tanned skin. | -2 | 0 | 0 | ** | ** |  |
| 1. I tan because tanned skin makes me feel closer to my ethnic origin. | -4 | -3 | -4 | * |  | ** |
| 1. I tan because then I get compliments on my appearance. | 1 | -1 | 2 |  | ** | *** |
| 1. I tan because I feel more attractive with tanned skin. | 2 | 1 | 4 |  | *** | *** |
| 1. **I tan because I look more athletic with tanned skin.** | **1** | **1** | **1** |  |  |  |
| 1. I tan because it makes my body look more muscular. | -3 | 0 | 0 | *** | *** |  |
| 1. I tan because it makes me more appealing to my partner. | -1 | -2 | 1 |  | * | *** |
| 1. I tan because it makes my skin look more even and clean. | 0 | 0 | 3 |  | *** | *** |
| 1. I tan because I get freckles. | -1 | -2 | -3 | ** | *** |  |
| 1. **I tan because tanned skin is considered desirable in our society.** | **0** | **0** | **0** |  |  |  |
| 1. I tan because it makes me look better. | 2 | 2 | 4 |  | ** | ** |
| 1. I tan because tanned skin increases my sex appeal. | -1 | -1 | 1 |  | ** | *** |
| 1. I tan because I look slimmer with tanned skin. | -2 | -1 | -1 | ** |  |  |
| 1. I tan because the people around me tan regularly and I want to fit in. | -3 | -1 | -2 | ** |  |  |
| 1. **I tan because the people around me think a tan is attractive.** | **0** | **-1** | **-1** |  |  |  |
| 1. I tan because the people around me think that tanned skin makes me look healthier. | 0 | 0 | 1 |  | * | ** |
| 1. I tan because I have been doing so since I was a child. | -1 | 0 | -3 | * | *** | *** |
| 1. I tan because my parents taught me from an early age that tanned skin is desirable. | -4 | -2 | -4 | *** |  | * |
| 1. **I tan because my environment says it is good for my health to do so.** | **0** | **-1** | **-2** |  |  |  |
| 1. **I tan because social media gives me the impression that tanned skin is desirable.** | **-2** | **-2** | **-1** |  |  |  |
| 1. **I tan because celebrities also have tanned skin.** | **-3** | **-3** | **-2** |  |  |  |
| 1. I tan because of acne or other skin diseases (e.g., neurodermatitis). | 0 | -3 | -2 | *** |  | *** |
| 1. I tan because tanned skin conceals my skin disease (e.g., acne). | -1 | -4 | 0* | *** | * | *** |
| 1. **I tan because I look healthier with tanned skin.** | **1** | **1** | **2** |  |  |  |
| 1. **I tan because vitamin D production through the sun is important to me.** | **3** | **2** | **3** |  |  |  |
| 1. I tan because my physician recommended me to go out in the sun. | -1 | -4 | -3 | *** | *** |  |
| 1. I tan because sunbathing is good for my mental health. | 4 | 4 | -1 |  | *** | *** |
| 1. I tan because the tanned skin is less sensitive to sunburn. | 0 | 1 | 1 | * |  |  |
| 1. I tan to strengthen my immune system. | 1 | 2 | 2 | * |  |  |
| 1. I tan because the sun gives me new energy. | 3 | 3 | -1 |  | *** | *** |

Items in bold = Consensus-Statements (none of the comparisons are significantly different)

Asterisk (*) = Distinguishing-Statements (Factor differs significantly from others), * p < 0.05, ** p < 0.01, *** p < 0.001
